# Utility of Generative Artificial Intelligence for Japanese Medical Interview Training: Randomized Crossover Pilot Study

**DOI:** 10.2196/77332

**Published:** 2025-08-01

**Authors:** Takanobu Hirosawa, Masashi Yokose, Tetsu Sakamoto, Yukinori Harada, Kazuki Tokumasu, Kazuya Mizuta, Taro Shimizu

**Affiliations:** 1 Department of Diagnostic and Generalist Medicine, Dokkyo Medical University, 880 Kitakobayashi, Mibu-cho, Shimotsuga, 321-0293, Japan, 81 282861111; 2 Department of General Medicine, Graduate School of Medicine, Dentistry and Pharmaceutical Sciences, Okayama University, Okayama, Japan; 3 Department of Intensive Care Medicine, Kameda Medical Center, Chiba, Japan

**Keywords:** artificial intelligence, generative artificial intelligence, medical interview training, mock patient, simulation education

## Abstract

**Background:**

The medical interview remains a cornerstone of clinical training. There is growing interest in applying generative artificial intelligence (AI) in medical education, including medical interview training. However, its utility in culturally and linguistically specific contexts, including Japanese, remains underexplored. This study investigated the utility of generative AI for Japanese medical interview training.

**Objective:**

This pilot study aimed to evaluate the utility of generative AI as a tool for medical interview training by comparing its performance with that of traditional face-to-face training methods using a simulated patient.

**Methods:**

We conducted a randomized crossover pilot study involving 20 postgraduate year 1‐2 physicians from a university hospital. Participants were randomly allocated into 2 groups. Group A began with an AI-based station on a case involving abdominal pain, followed by a traditional station with a standardized patient presenting chest pain. Group B followed the reverse order, starting with the traditional station for abdominal pain and subsequently within the AI-based station for the chest pain scenario. In the AI-based stations, participants interacted with a GPT-configured platform that simulated patient behaviors. GPTs are customizable versions of ChatGPT adapted for specific purposes. The traditional stations involved face-to-face interviews with a simulated patient. Both groups used identical, standardized case scenarios to ensure uniformity. Two independent evaluators, blinded to the study conditions, assessed participants’ performances using 6 defined metrics: patient care and communication, history taking, physical examination, accuracy and clarity of transcription, clinical reasoning, and patient management. A 6-point Likert scale was used for scoring. The discrepancy between the evaluators was resolved through discussion. To ensure cultural and linguistic authenticity, all interviews and evaluations were conducted in Japanese.

**Results:**

AI-based stations scored lower across most categories, particularly in patient care and communication, than traditional stations (4.48 vs 4.95; *P*=.009). However, AI-based stations demonstrated comparable performance in clinical reasoning, with a nonsignificant difference (4.43 vs 4.85; *P*=.10).

**Conclusions:**

The comparable performance of generative AI in clinical reasoning highlights its potential as a complementary tool in medical interview training. One of its main advantages lies in enabling self-learning, allowing trainees to independently practice interviews without the need for simulated patients. Nonetheless, the lower scores in patient care and communication underline the importance of maintaining traditional methods that capture the nuances of human interaction. These findings support the adoption of hybrid training models that combine generative AI with conventional approaches to enhance the overall effectiveness of medical interview training in Japan.

## Introduction

### Medical Interview Training

Medical interview training is an essential part of medical education, significantly influencing clinical competence, patient satisfaction, and treatment outcomes [1-5]. Effective medical interviewing skills are crucial not only for accurate diagnosis but also for establishing trust and rapport among health care professionals, patients, and their families [6-11]. For example, several studies revealed that proper diagnoses can often be made based mainly on an effective medical interview rather than investigations [12,13]. These findings highlighted the pivotal role of communication skills in clinical practice.

### Barriers to Medical Interview Training

Despite its importance, medical interview training often faces several barriers [14]. For instance, traditional training methods typically involve simulated patient interactions, which are resource-intensive, requiring substantial time commitments from both medical trainees and educators [15]. While simulation training can provide valuable experiential learning [16-18], its scalability is often limited by resource and financial constraints [19-22]. Consequently, medical students and junior physicians may not receive sufficient opportunities for comprehensive and repeated practice, limiting their development of essential communication and clinical reasoning skills [23,24].

### Potential of Artificial Intelligence for Medical Interview Training

In response to these challenges, artificial intelligence (AI) has emerged as a promising tool in medical education [25-28]. Until recent breakthroughs, AI performance remained inadequate due to technical limitations [29]. However, the current development of suitable technologies, including Compute Unified Device Architecture and advanced graphics processing units, has remarkably enhanced AI capabilities [30-33]. AI-driven platforms offer scalable, consistent, and flexible training experiences that allow trainees to practice extensively [34]. These tools have the potential to bridge gaps in access to traditional training by enabling frequent, independent practice [35,36].

### Potential of Generative AI for Medical Interview Training

Generative AI, a subset of AI that generates human-like responses and interactions [37,38], presents exciting potential for medical interview training [39,40]. It often incorporates natural language processing and large language models, which enable it to generate and respond to human dialogue in contextually appropriate ways [41,42]. Unlike traditional training methods, generative AI can simulate diverse and complex patient scenarios, providing interactive, responsive, and personalized feedback [43]. This capability not only enhances clinical reasoning but also facilitates self-learning, allowing students to practice repeatedly at their convenience [44-46].

### Prior Work

Recent studies have explored the application of generative AI in medical interview training, particularly in the context of Objective Structured Clinical Examinations (OSCEs). For example, research in Japan reported that GPT-4 (legacy) based stations outperformed traditional stations for medical students [47]. However, direct comparison with previous work is limited by differences in AI versions, participant populations, clinical cases, and study designs. Further, earlier studies found that previous versions of GPT occasionally generated implausible responses [48,49]. Additionally, the comparative performance between ChatGPT-4 (legacy) and human physicians in conducting medical interviews revealed comparable aggregate scores across 5 components on the 5-Likert scale (15/25 vs 15/25; *P*<.28) [50].

### Research Gap and Aim of the Study

Despite these advances, there is still a lack of research evaluating the utility of generative AI tools in Japanese clinical contexts. Cultural and linguistic nuances, including Japanese, play a significant role in effective communication [51-53]. However, there is a lack of enough research evaluating the effectiveness and adaptability of generative AI tools within the Japanese clinical context. To the best of our knowledge, there is limited research regarding the effectiveness and applicability of generative AI-driven training tools for Japanese medical trainees [47]. Therefore, this study aimed to evaluate the utility and limitations of generative AI by comparing AI-driven medical interview scenarios with traditional mock patient interactions among postgraduate physicians in Japan.

## Methods

### Setting

This pilot study was conducted in the Department of Diagnostic and Generalist Medicine (general internal medicine [GIM]) at Dokkyo Medical University, Tochigi, Japan.

To minimize variability in participants’ medical interview skills, a randomized crossover design was used [54]. All interviews and evaluations were conducted in Japanese to preserve cultural and linguistic integrity. The study consisted of 3 main components: participant recruitment, medical interview implementation, and interview evaluation. This study adhered to the CONSORT-EHEALTH (Consolidated Standards of Reporting Trials of Electronic and Mobile Health Applications and Online Telehealth) guidelines (the CONSORT-EHEALTH checklist is provided in Checklist 1).

### Ethical Considerations

Ethics approval was obtained from the Institutional Review Board at Dokkyo Medical University Hospital (number R-79‐14J). The research adhered strictly to the Helsinki Declaration guidelines to ensure ethical conduct in human participant research.

### Participant Inclusion

Participants included postgraduate year 1‐2 physicians rotating through the GIM department at Dokkyo Medical University Hospital between April 2024 and January 2025. All eligible physicians during this period were invited to participate. Exclusion criteria included hearing loss or unwillingness to attend the research. Before enrollment, all participants received detailed explanations regarding the study’s objectives, procedures, and confidentiality protocols from researchers. Written informed consent was obtained from each participant.

### Medical Interview

#### Overview

Participants were randomly allocated into 2 groups through block randomization to ensure an equal group size [55]. The random allocation sequence was generated by an independent researcher (KM) using Microsoft Excel. This ensured balanced distribution and minimized potential confounding from individual differences.

Each participant completed 2 types of medical interview stations—an AI-based station using the GPTs platform and a traditional station with face-to-face interviews with a trained actor simulating the patient (simulated patient). The 2 stations covered separate clinical cases: abdominal pain and chest pain. In the AI-based stations, participants typed their questions and responses into a laptop computer to interact with the GPTs platform. In the traditional stations, participants engaged in spoken conversation with a simulated patient to conduct the medical interview.

Participants in Group A started with the AI-based interview on abdominal pain, followed by the traditional interview on chest pain. Group B began with the traditional interview on abdominal pain and proceeded to the AI-based interview on chest pain.

#### Station Structure

Both the AI-based and traditional stations followed an identical structure based on The OSCE [56]. Initially, participants reviewed the simulated patient’s basic information for 1 minute. The medical interview, including questions relevant to physical examination, was conducted over 15 minutes. Physical examinations were not actually performed in either station due to maintaining consistency with the text-based interaction in the AI-based station. Following the medical interview, participants had 6 minutes to formulate an assessment and plan. Brief feedback and learning points were then provided for several minutes, after which the participants moved to the next station.

#### GPTs Setting

GPTs are custom versions of ChatGPT that we can adjust for a specific purpose without programming [57]. In this study, the systems were configured to simulate a patient based on detailed case information provided in Japanese. Importantly, the GPTs were not trained or fine-tuned in the Japanese medical language. The systems did not provide a final diagnosis, even if participants asked. Furthermore, if a participant inputted medical jargon [58], GPTs responded with queries such as “What is XXX?” to simulate realistic patient confusion. Additional configuration with translation in English details is provided in Multimedia Appendix 1.

#### Simulated Patient

The traditional simulated patient interviews were conducted by researcher TH, who was trained to ensure consistency in responses and demeanor. This approach was chosen because the researcher serves not only as a trained actor simulating symptoms but also as an educator providing brief feedback to the participants at the end of each session. Identical clinical scenarios were used across both groups, based on a widely used and standardized textbook for medical interview training [59].

### Evaluation for Medical Interview

Traditional stations were video-recorded and transcribed. AI-based stations used the saved text logs. For consistency in evaluation, the transcriptions were refined to match the same structures between stations. For example, headers labeled as “GPTs” in the AI-based stations were changed to “Patient.” Self-introduction parts were removed. The corresponding text files were also anonymized. Sample transcript with translation in English is available in Multimedia Appendix 2.

Two experienced physicians, MK and TSa, independently evaluated the transcripts. The evaluators did not take part in the previous participant recruitment and medical interview implementation. Evaluators used a structured scoring system using a 6-point Likert scale, where 1 is inferior and 6 is excellent. Assessments were based on six key domains: (1) patient care and communication skills, (2) thoroughness of history-taking, (3) physical examination proficiency, (4) accuracy and clarity of transcription, (5) clinical reasoning capability, and (6) overall patient management strategies. The discrepancy was resolved through discussion. Evaluators were blinded to interview methods and participant identity. They assessed transcripts in random order. The scoring system is also based on The OSCE [56,60].

### Statistical Analysis

#### Outcome

The primary outcomes were the comparison of mean scores between AI-based and traditional stations for the whole and each assessment component. The secondary outcome measures involved comparisons within each clinical case, abdominal pain, and chest pain, by interview style.

#### Data Collection

Baseline characteristics data were collected, including years since obtaining a degree in medicine and sex. All medical interviews were also recorded to ensure accurate transcription: traditional stations were video-recorded, and AI-based stations preserved the conversation logs as text.

#### Analysis

For both primary and secondary outcomes, scores on the 6-point Likert scale were presented as mean with 95% CIs. To assess the appropriateness of statistical tests, the normality of the paired score differences between AI-based and traditional stations was checked using the Shapiro-Wilk test [61]. As the score differences were not normally distributed, the Mann-Whitney *U* test was used as the primary method for comparing paired outcomes between the 2 stations. A *P* value <.05 was considered statistically significant. For reference, the 95% CIs are provided to supplement the *P* values (Multimedia Appendix 3 contains detailed normality test results and detailed mean difference).

Continuous variable related to participant characteristics is presented as medians and IQRs and compared using the Mann-Whitney *U* test. The categorical variable was compared using the Fisher exact test. All statistical analyses were conducted using R (version 4.2.2; The R Foundation for Statistical Computing) for MacOS X.

## Results

### Participants Characteristics

**Figure 1.**
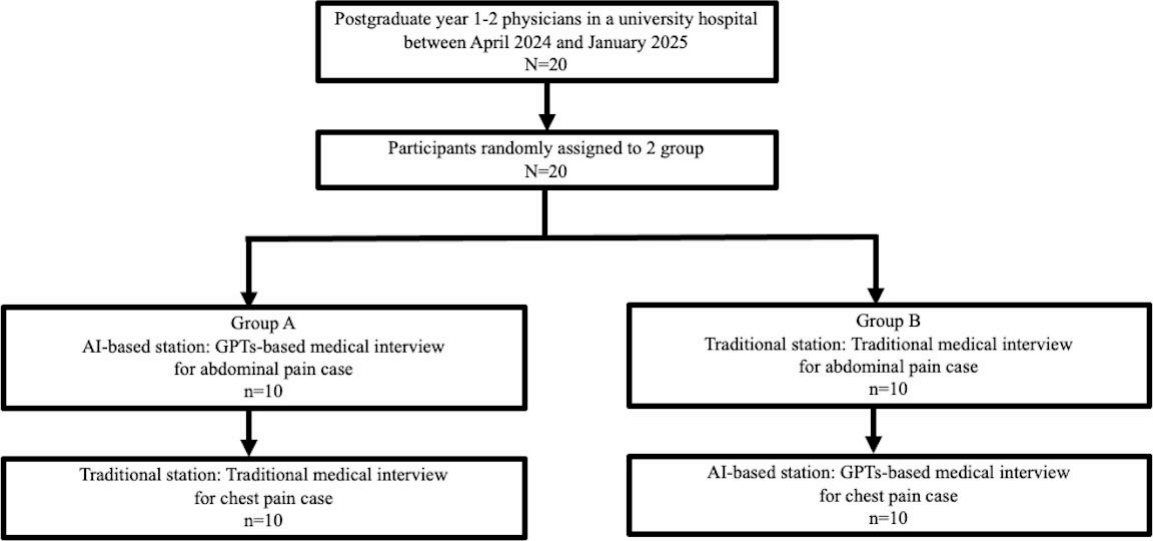
The flow chart includes participants and allocating the groups.

A total of 20 postgraduate physicians were enrolled (Figure 1). Among them, 11 (56%) physicians were first year after graduation, while 9 (45%) physicians were in their second year. Two (10%) female participants were included. There were no statistical differences in participant characteristics between group A and group B, as shown in Table 1.

**Table 1.** Participants' characteristics.

Variable	Group A (N=10)	Group B (N=10)	*P* value
Female, n (%)	0 (0)	2 (20)	.47^a^
Years after graduation (years), median (IQR)	1.5 (1.0)	1.0 (1.0)	.69^b^

a Fisher exact test.

b Mann-Whitney *U* test.

### Evaluation Outcomes

Performance scores were compared between the AI-based and traditional stations across overall and 6 assessment domains, as shown in Table 2. Overall, the total score was 4.89 in the AI-based stations compared with 5.47 in the traditional stations (*P*<.001).

**Table 2.** Performance scores were compared between the artificial intelligence–based and traditional stations across overall and 6 assessment domains.

Scoring system with a 6-point Likert scale	Artificial intelligence–based (GPTs) stations (N=20^a^), 95% CI	Traditional stations (N=20^a^), 95% CI	*P* value^b^
Overall	4.89 (4.74‐5.04)	5.47 (5.35‐5.58)	<.001
Patient care and communication	5.05 (4.73‐5.37)	5.45 (5.06‐5.84)	.04
History taking	4.90 (4.69‐5.11)	5.30 (4.96‐5.65)	.04
Physical examination	5.10 (4.73‐5.47)	5.80 (5.61‐5.99)	.001
Accuracy and clarity of transcription	4.70 (4.36‐5.05)	5.40 (5.16‐5.64)	.002
Clinical reasoning	4.75 (4.23‐5.27)	5.30 (4.96‐5.64)	.13
Management	4.85 (4.34‐5.36)	5.55 (5.31‐5.79)	.02

a Crossover participants with 10 chest paincasese and 10 abdominal paincasese.

b Mann-Whitney *U* test.

AI-based stations yielded slightly lower scores in patient care and communication (mean score: 5.05 vs 5.45; *P*=.04). Scores in other domains such as history taking (4.90 vs 5.30; *P*=.04), physical examination (5.10 vs 5.80*; P*=.001), accuracy and clarity of transcription (4.70 vs 5.40; *P*=.002), and management (4.85 vs 5.55; *P*=.02) also trended lower for the AI-based stations. In contrast, the domain of clinical reasoning showed no significant difference between AI-based and traditional stations (4.75 vs 5.30; *P*=.13).

### Subgroup Analysis

#### Overview

Subgroup analyses were performed to compare the AI-based and traditional stations for each clinical case individually. The initial case presented to participants was abdominal pain, followed sequentially by a chest pain case.

#### Abdominal Pain Cases

For the abdominal pain case, as shown in Table 3, the overall score was significantly lower in the AI-based stations compared with the traditional stations (4.70 vs 5.48; *P*<.001). Notably, scores for clinical reasoning (4.30 vs 5.50; *P*=.01) and accuracy and clarity of the transcript (4.40 vs 5.40; *P*=.009) were significantly lower in the AI-based stations. While other domains such as patient care and communication (5.00 vs 5.60; *P*=.06), physical examination (5.20 vs 5.80; *P*=.06), and management (4.60 vs 5.50; *P*=.07) were lower in the AI-based stations than the traditional stations, these did not reach statistical significance.

**Table 3.** Subgroup analysis for abdominal pain cases compared the artificial intelligence-based and traditional stations across overall and 6 assessment domains.

Scoring system with a 6-point Likert scale	Artificial intelligence-based (GPTs) stations (N=10), 95% CI	Traditional stations (N=10), 95% CI	*P* value^a^
Overall	4.70 (4.47‐4.93)	5.48 (5.31‐5.66)	<.001
Patient care and communication	5.00 (4.52‐5.48)	5.50 (4.80‐6.20)	.06
History taking	4.70 (4.35‐5.05)	5.20 (4.54‐5.86)	.17
Physical examination	5.20 (4.64‐5.76)	5.80 (5.50‐6.10)	.06
Accuracy and clarity of transcription	4.40 (3.78‐5.00)	5.40 (5.03‐5.77)	.009
Clinical reasoning	4.30 (3.54‐5.06)	5.50 (5.12‐5.88)	.01
Management	4.60 (3.70‐5.50)	5.50 (5.12‐5.88)	.07

a Mann-Whitney *U* test.

#### Chest Pain Cases

In the case of chest pain, as shown in Table 4, the AI-based stations scored slightly lower in overall scores compared with those in the traditional stations (5.08 vs 5.45; *P*=.004). Physical examination skills were also significantly lower in the AI-based stations (5.00 vs 5.80; *P*=.009). Other domains, including patient care and communication (5.10 vs 5.40; *P*=.37), history taking (5.10 vs 5.40; *P*=.14), and transcription clarity (5.00 vs 5.40; *P*=.09), demonstrated trends in favor of the traditional stations but did not reach significance. Clinical reasoning scores were comparable between the 2 stations (5.10 vs 5.20; *P*=.72), indicating consistent reasoning performance regardless of the interview modality.

**Table 4.** Subgroup analysis for chest pain cases compared the artificial intelligence–based and traditional stations across overall and 6 assessment domains.

Scoring system with a 6-point Likert scale	Artificial intelligence-based (GPTs) stations (N=10), 95% CI	Traditional stations (N=10), 95% CI	*P* value^a^
Overall	5.08 (4.90‐5.27)	5.45 (5.29‐5.61)	.004
Patient care and communication	5.10 (4.57‐5.63)	5.40 (4.90‐5.90)	.37
History taking	5.10 (4.87‐5.33)	5.40 (5.03‐5.77)	.14
Physical examination	5.00 (4.42‐5.58)	5.80 (5.50‐6.10)	.009
Accuracy and clarity of transcription	5.00 (4.66‐5.34)	5.40 (5.03‐5.77)	.09
Clinical reasoning	5.20 (4.46‐5.94)	5.10 (4.47‐5.73)	.72
Management	5.10 (4.47‐5.73)	5.60 (5.23‐5.97)	.20

a Mann-Whitney *U* test.

## Discussion

### Principal Findings

This study evaluated the utility of generative AI in medical interview training compared with traditional simulated patient interactions among postgraduate physicians in Japan. The principal findings indicate that while AI-based stations provide alternative training methods, they generally yield lower performance scores across several critical domains, including patient care and communication, thoroughness of history-taking, physical examination proficiency, accuracy and clarity of transcription, and management. Participants may have found it difficult to express empathy or engage in natural conversation through typed exchanges [62], limiting the development of interpersonal skills in the GPT stations. While generative AI demonstrates the potential for medical interview training, our findings suggest that it is best suited as a supplementary tool rather than a replacement for traditional simulated patient interactions. The lower performance observed in domains dependent on human interaction—such as communication and patient care—highlights current limitations in AI’s ability to simulate empathy and nonverbal cues. Traditional stations, facilitated by trained actors or simulated patients, remain essential for developing advanced interpersonal and communication skills.

A key methodological aspect of this study was configuring the GPT instance to realistically simulate Japanese patient interactions. The GPTs were set up to operate entirely in Japanese, with patient cases, and presented in culturally appropriate language. To enhance authenticity, the system was instructed to respond using typical expressions. Furthermore, the GPTs were directed to avoid using medical terminology.

Despite the limitations in interpersonal skill development, domains such as clinical reasoning remained comparable between GPTs and traditional stations. This finding reinforces the potential of AI-based stations in supporting cognitive aspects of clinical assessment. This result highlights the enduring value of traditional stations, where human dynamics and emotional responsiveness can be authentically practiced and assessed.

Subgroup analyses further demonstrated these differences across specific clinical scenarios. In the abdominal pain case, AI-based stations scored significantly lower in overall performance, clinical reasoning, and transcription clarity. Although other domains like patient care and physical examination were also lower, they did not reach statistical significance. For the chest pain case, while the overall scores were also lower in the GPT stations, the difference was narrower, with physical examination skills showing the most significant disparity. Interestingly, a sub-analysis of abdominal pain cases revealed a significantly lower clinical reasoning score in the AI-based station. This disparity may be attributed to differences in case complexity or the broader differential diagnoses associated with abdominal presentations. In particular, abdominal pain may demand a nuanced interpretation of information [63], suggesting that the limited interactivity of the AI-based format may have constrained diagnostic reasoning. This finding, which was not apparent in the overall analysis, provides an important supplementary insight. It highlights the need to account for case-specific characteristics when selecting cases or designing AI-driven educational tools [64].

### Limitations

Several limitations must be acknowledged. First, this study was designed as a feasibility and exploratory trial and was not fully powered or intended for formal hypothesis testing. The small sample size (n=20) and limited number of stations constrain the generalizability of the findings. The primary goal was to assess the feasibility and gather preliminary data to inform future larger-scale studies. Second, the study only included postgraduate physicians from a single institution, potentially restricting the diversity and representativeness of the findings. Results may not be directly applicable to undergraduate medical students, other health care professionals, or participants from different institutions or backgrounds. Third, the mode of interaction differed between AI, typed input, and traditional stations, spoken conversation, which may have inherently biased communication-related scores. Furthermore, physical examinations were not really performed in either station to unify the format for the text-based interaction in the AI-based station, which could have influenced how this domain was assessed. Fourth, the blinded evaluators may have been able to discern the interview modality indirectly, potentially introducing bias. Fifth, it should also be noted that there was some difference in difficulty between the abdominal pain and chest pain cases. This discrepancy arose because it is inherently challenging to create cases of identical complexity based on different primary concerns. Such differences in case difficulty may have influenced performance results and should be considered when interpreting subgroup analyses. Finally, the study was conducted in a single language using only one generative AI platform, GPTs, limiting its applicability to other languages, cultural contexts, and AI technologies.

### Comparison With Prior Work

The current findings expand upon the existing literature. Previous research on OSCEs in Japan found that GPT-4 (legacy) based stations outperformed traditional stations of medical students, with significantly higher total scores across 5 components of a 6-point Likert scale (28.1/31, vs 27.1/31; *P*=.01) [47]. Several differences between the previous study and the current findings limit direct comparison. These include variations in the AI versions used (GPT-4 legacy vs GPTs), participant demographics (medical students vs physicians), cases, and study design (nonrandomized vs randomized crossover).

In relation to the quality of simulated patient responses, previous research on GPT-3.5 and GPT-4 (legacy) indicated implausible response rates of 2% (14/842) and 0.7% (13/1894), respectively [48,49]. In this study using the latest GPTs, responses were almost entirely plausible, with only one instance where GPTs prematurely revealed full physical exam results. This highlights rare but relevant issues in prompt sensitivity.

These findings are particularly promising for resource-limited settings or educational scenarios where access to trained professionals for mock interviews is constrained [65]. However, caution remains warranted in extrapolating these outcomes to real-world clinical environments.

### Future Direction

To expand the utility of generative AI in medical interview training, future research should aim for broader validation across diverse educational settings, languages, and digital technology platforms. Improvements in multimodal AI and the integration of voice-based interactions may enhance the realism and interpersonal aspects of AI simulations [66]. Multimodal AI processes and understands information from different types of data, including text, images, audio, video, and sometimes even sensor data [67]. Future investigations should also explore the longitudinal impacts of repeated practice with AI-driven tools to better evaluate the long-term benefits [68]. Additionally, studies comparing hybrid models—such as AI-assisted interviews followed by human debriefing—may offer insights into how best to combine the strengths of both methods [69,70].

### Conclusions

This study provides important proof-of-concept evidence for the use of generative AI, specifically GPTs, as a tool in medical interview training among postgraduate physicians. While the AI-based (GPT) station underperformed compared with traditional stations across several domains, including patient care and communication, the performance in clinical reasoning was comparable. These results suggested that generative AI could serve as a supplemental tool for medical education in cognitive components of clinical assessment.

The practical implications for medical education are important. Generative AI can enable self-directed, scalable, and accessible medical interview practice. However, the current findings also reinforce the value of human interaction in developing nuanced communication and empathy. Therefore, the adoption of hybrid educational models may be particularly effective. This approach is the unique strength of combining AI and human educators in simulation-based learning environments.

Nevertheless, these conclusions are preliminary. The small sample size, single-institution setting, and limited number of clinical cases restrict the generalizability of our findings. The crossover design, differences in case complexity, modality of interaction (typed vs spoken), and the use of a single AI language model and language all further limit broad application. These feasibility findings warrant cautious interpretation and highlight the need for larger, multicenter, and longitudinal studies to establish comparative effectiveness and assess the long-term educational impact of AI-assisted training.

Future research should explore the integration of multimodal AI systems to enhance the realism and authenticity of patient simulations. Additionally, multiple institutional collaborations, broader participant demographics, and studies in other languages and contexts are needed to determine the true potential and limitations of AI in medical education.

## Supplementary material

10.2196/77332 Multimedia Appendix 1 Details of GPTs setting for artificial intelligence (AI)–based medical interview training.

10.2196/77332 Multimedia Appendix 2 An example of transcription.

10.2196/77332 Multimedia Appendix 3 Supplementary statistical analysis.

10.2196/77332 Checklist 1 CONSORT-EHEALTH (Consolidated Standards of Reporting Trials of Electronic and Mobile Health Applications and Online Telehealth) checklist.

## Abbreviations

AI: artificial intelligence
CONSORT-EHEALTH: Consolidated Standards of Reporting Trials of Electronic and Mobile Health Applications and Online Telehealth
GIM: general internal medicine
OSCE: Objective Structured Clinical Examination
