# Supplementary material for: Utility of Generative Artificial Intelligence for Japanese Medical Interview Training: Randomized Crossover Pilot Study
Source: JMIR Med Educ. 2025 Aug 1;11:e77332. doi: 10.2196/77332 (PMC12316404; doi:10.2196/77332)
Supplement: Multimedia Appendix 2 [file mededu-v11-e77332-s002.docx]

Table S2. The example of transcription.

Physician: Hello, we will start the interview now.

Patient: Hello.

Physician: You mentioned you have chest pain. Could you explain this event in chronological order, please?

Patient: I don't understand. Please ask a more specific question.

Physician: When did the chest pain start?

Patient: It started 50 minutes ago.

Physician: Was the onset of the chest pain sudden or gradual?

Patient: It was sudden.

(The medical interview was continued)
