# Supplementary material for: Utility of Generative Artificial Intelligence for Japanese Medical Interview Training: Randomized Crossover Pilot Study
Source: JMIR Med Educ. 2025 Aug 1;11:e77332. doi: 10.2196/77332 (PMC12316404; doi:10.2196/77332)
Supplement: Multimedia Appendix 3 [file mededu-v11-e77332-s003.docx]

Table S3. Supplementary Statistical Analysis

**Normality Assessment (Shapiro-Wilk Test)**

| **Comparison** | **W Statistic** | ***P* value** | **Interpretation** |
| --- | --- | --- | --- |
| AI-based (GPTs) stations | 0.837 | <.001 | Not normally distributed |
| Traditional stations | 0.726 | <.001 | Not normally distributed |
| Difference (AI - Traditional) | 0.908 | <.001 | Not normally distributed |

**Paired Difference Analysis**

Paired t-test for reference only

t = -6.138

degrees of freedom = 119

*P* <.001

Mean difference = -0.575

95% Confidence Interval: [-0.760, -0.390]
